
JOURNAL INFORMATION
==============================
NLM Title Abbreviation: ChemistryOpen
Journal ID: ChemistryOpen
NLM Journal ID: 101594811
Journal ID: open

ChemistryOpen

EISSN: 2191-1363
Publisher: Wiley

ARTICLE INFORMATION
==============================
PMCID: PMC3703808
PMID: 24551540
DOI: 10.1002/open.201390011
Article version: 1
Subjects: Graphical Abstract

Graphical Abstract: ChemistryOpen 3/2013

Print publication date: 2013 Jun
Electronic publication date: 2013 Jun 20
Volume: 2
Issue: 3
First page: 79
Last page: 82
Copyright: Copyright © 2013 WILEY-VCH Verlag GmbH & Co. KGaA, Weinheim
Copyright year: 2013
License: Re-use of this article is permitted in accordance with the Creative Commons Deed, Attribution 2.5, which does not permit commercial exploitation.
License URL: https://creativecommons.org/licenses/by/2.5/

==============================
ChemistryOPEN
Including Thesis Treasury

03/2012

ChemistryOPEN... is a multidisciplinary, gold-road, open-access, international forum for the publication of Full Papers and Communications from all areas of chemistry and related fields. *ChemistryOpen* also publishes the Thesis Treasury containing summaries of Ph.D. theses and links to the full version via our homepage. Based in Europe, *ChemistryOpen* attracts authors and readers from around the world, as open-access publishing becomes more important in all areas of chemistry. *ChemistryOpen* is co-owned by ChemPubSoc Europe and published by Wiley-VCH. Authors can submit their primary research articles and thesis summaries via our homepage by clicking "submit an article". All contributions considered suitable for publication are subject to peer review, and if accepted, electronically processed and published online ensuring high quality and short publication times.

Discover this journal online at:
WILEY ONLINE LIBRARY
wileyonlinelibrary.com

Most of the articles in this issue have already appeared online on wileyonlinelibrary.com. See http://www.chemistryopen.org under EarlyView®

### COVER PICTURE

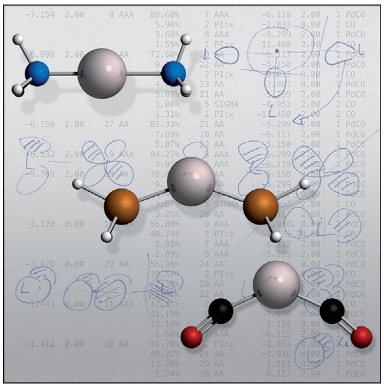

**The cover picture illustrates** the authors' quantum chemical finding that π electrons can significantly bend otherwise linear $d^{10}$-$ML_2$ complexes through backbonding. The foreground features a typical series of linear and nonlinear computed equilibrium geometries, while, in the background, one discerns quantitative numerical output of the bonding and energy decomposition analyses (EDA). The interpretation of the numerical data in terms of Kohn–Sham molecular orbital (MO) theory constitutes a predictive bonding model that explains the effects. The essence of this model has been sketched on paper during a brainstorming session (see the blue, hand-drawn orbital diagrams). It reveals that the second π-accepting ligand is, in a sense, hunting for "fresh" (not yet stabilized) $d_\pi$ electrons. The insights obtained in this work are relevant not only for structural coordination chemistry but are envisaged to lead to applications in rational catalyst design. For more details, see the Full Paper by F. Matthias Bickelhaupt et al., on p. 106 ff.

### NEWS

Spotlights on our sister journals *84–87*

© 2013 Wiley-VCH Verlag GmbH & Co. KGaA, Weinheim

ChemistryOPEN Masthead
ChemPubSoc Europe
www.chemistryopen.org

Austria
Belgium
Czech Republic
France
Germany
Greece
Hungary
Italy
Netherlands
Poland
Portugal
Slovakia
Spain
Sweden
Switzerland

### Editorial Advisory Board

### *Chairmen*

**Ramón Martínez-Máñez (Spain)**
**Thomas Wirth (UK)**

### *Honorary Chairman*

**Jean-Marie Lehn (France)**

### *Members*

Fernando Albericio (Spain)
Valentin Ananikov (Russia)
Didier Astruc (France)
Jochen Autschbach (USA)
Martin Banwell (Australia)
Vincenzo Barone (Italy)
F. Matthias Bickelhaupt (The Netherlands)
Kathrin Breuker (Austria)
Young Keun Chung (South Korea)
Richard G. Compton (UK)
Ulf Diederichsen (Germany)
Lothar Dunsch (Germany)
Antony Fairbanks (New Zealand)
Ernest Giralt (Spain)
Stefan Grimme (Germany)
Dirk Guldi (Germany)
Akira Harada (Japan)
Narayan Hosmane (USA)
Ken Houk (USA)
Osamu Ito (Japan)
Christoph Janiak (Germany)
Kazunori Kataoka (Japan)
Yoshiaki Kiso (Japan)
Mats Larhed (Sweden)
Lechoslav Latos-Grażyński (Poland)
Thisbe Lindhorst (Germany)
Luis M. Liz-Marzán (Spain)
Carlos Lodeiro (Portugal)
Todd L. Lowary (Canada)
Jean Martinez (France)
Pedro Matias (Portugal)
Stefan Matile (Switzerland)
Pedro Molina (Spain)
K. C. Nicolaou (USA)
Berit Olofsson (Sweden)
Oliver Reiser (Germany)
Kari Rissanen (Finland)
Knut Rurack (Germany)
James Rusling (USA)
Albert Schenning (The Netherlands)
Robert Schlögl (Germany)
Patrick Schmuki (Germany)
Ulrich S. Schubert (Germany)
Min Shi (China)
Norio Shibata (Japan)
Rint Sijbesma (The Netherlands)
Choong Eui Song (South Korea)
Petr Štěpnička (Czech Republic)
Brian Stoltz (USA)
Steve Suib (USA)
Reshef Tenne (Israel)
Matthew Todd (Australia)
Claudio Toniolo (Italy)
Jaume Veciana (Spain)
Hans-Achim Wagenknecht (Germany)
Ralph Weissleder (USA)
Gunther Wittstock (Germany)
Vivian W. W. Yam (China)
Chun-Hua Yan (China)
Jincai Zhao (China)

**Open Access and Copyright**

All research articles published by *ChemistryOpen* are fully open access: immediately freely available to read, download and share. All *ChemistryOpen* articles are published under the terms of the Creative Commons Attribution Non-Commercial License which permits use, distribution and reproduction in any medium, provided the original work is properly cited and is not used for commercial purposes.

Copyright on any research article published by *ChemistryOpen* is retained by the author(s). Authors grant Wiley a license to publish the article and identify itself as the original publisher. Authors also grant any third party the right to use the article freely as long as its integrity is maintained and its original authors, citation details and publisher are identified.

**Use by commercial “for-profit” organisations**

Use of Wiley Open Access articles for commercial, promotional, or marketing purposes requires further explicit permission from Wiley and will be subject to a fee. Copyright 2012 Wiley-VCH Verlag GmbH & Co. KGaA, Weinheim. All rights reserved. Requests to reproduce material from this journal content should be addressed to rights@wiley-vch.de, and reprints of *ChemistryOpen* articles can be purchased from chem-reprints@wiley-vch.de.

**Disclaimer**

The Publisher and Editors cannot be held responsible for errors or any consequences arising from the use of information contained in this journal; the views and opinions expressed do not necessarily reflect those of the Publisher and Editors, neither does the publication of advertisements constitute any endorsement by the Publisher and Editors of the products advertised.

Wiley Open Access articles posted to repositories or websites are without warranty from Wiley of any kind, either express or implied, including, but not limited to, warranties of merchantability, fitness for a particular purpose, or non-infringement. To the fullest extent permitted by law Wiley disclaims all liability for any loss or damage arising out of, or in connection with, the use of or inability to use the content.

**Editors:**
Karen Hindson, Haymo Ross

**Deputy Editor:**
Natalia Ortúzar

**Associate Editor:**
Saskia Neubacher

**Secretary:**
Vanessa Müller

**Production:**
Stefanie Klein, Margitta Schmitt

**Telephone:**
(+ 49) 6201-606-305

**Fax:**
(+ 49) 6201-606-203

**E-mail:**
chemistryopen@wiley-vch.de

**Courier Services:**
Boschstrasse 12
69469 Weinheim (Germany)

**Regular Mail:**
Postfach 10 11 61
69451 Weinheim (Germany)

**Homepage:**
http://www.chemistryopen.org

**Manuscript Submission & Personal Homepage**
manuscriptXpress
**http://www.manuscriptxpress.com**

**Customer Services:**
www.wileycustomerhelp.com

**Marketing:**
Monika Silz (ext. 458, fax: ext. 100,
e-mail: msilz@wiley-vch.de)

**Advertising:**
Marion Schulz (ext. 565, fax: ext. 550,
e-mail: mschulz@wiley-vch.de, jspiess@wiley-vch.de)

**Copyright Permissions:**
Bettina Loycke (ext. 280, fax: ext. 447,
e-mail: rights@wiley-vch.de)

**Reprints, e-prints, posters:**
Carmen Leitner (fax: ext. 226)
e-mail: chem-reprints@wiley-vch.de

**Publishers:**
WILEY-VCH Verlag GmbH & Co. KGaA,
Postfach 10 11 61
69451 Weinheim (Germany)
http://www.wiley-vch.de/

**Publication:**
6 issues in 2013

© 2013 WILEY-VCH Verlag GmbH & Co. KGaA, Weinheim.

### COVER PROFILE

"*Being able to compute what molecules do is nice. But a more fundamental understanding of why this is so—for example, why they adopt the geometry they have or why they possess a particular reactivity—constitutes the true challenge and the ultimate objective of my profession.*" More about the story behind the cover research can be found on p. 106.

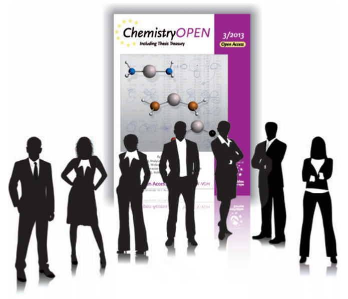

*L. P. Wolters, F. M. Bickelhaupt**

***78 – 78***

**Nonlinear $d^{10}$-$ML_2$ Transition-Metal Complexes**

### COMMUNICATIONS

**The sum of its parts:** Nanocomposites of $Fe^{10}BO_3/Fe_3O_4/SiO_2$ and $GdFeO_3/Fe_3O_4/SiO_2$, synthesized by a gel combustion technique using citric acid as fuel and chelation, exhibited strong magnetic properties indicating that they might have potential applications in diagnostic analysis of cancer through use in neutron capture therapy.

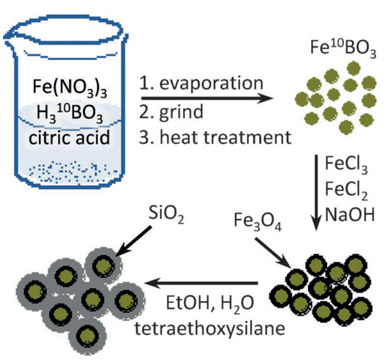

*S. Gao, X. Liu, T. Xu, X. Ma, Z. Shen, A. Wu, Y. Zhu, N. S. Hosmane**

***88 – 92***

**Synthesis and Characterization of $Fe^{10}BO_3/Fe_3O_4/SiO_2$ and $GdFeO_3/Fe_3O_4/SiO_2$: Nanocomposites of Biofunctional Materials**

### FULL PAPERS

**Glowing sandwich!** Self-organized $TiO_2$-nanotube layers can be used for immunoassay-type sensing. $TiO_2$ nanoparticles coated with CdTe quantum dots are used as signal amplification elements. We show that this, in combination with the advantages of a $TiO_2$-nanotube electrode, leads to an outstanding detection limit in electrochemiluminescent techniques.

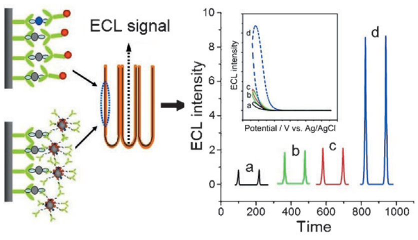

*Z.-D. Gao, Q.-L. Zhuang, Y.-Y. Song,* K. Lee, P. Schmuki**

***93 – 98***

**Signal Amplification Strategy Based on $TiO_2$-Nanotube Layers and Nanobeads Carrying Quantum Dots for Electrochemiluminescent Immunosensors**

**Choices, choices:** A conjugate of cyclam and the matrix metalloproteinase (MMP) inhibitor marimastat was synthesized to give a compound with two potential zinc-binding groups (ZBGs). The first equivalent of Cu(II) or Zn(II) was chelated by the cyclam-triazole moiety rather than the hydroxamic acid. Both the conjugate and its metal complexes exhibited potent and selective MMP inhibitory activity, and displayed no cytotoxicity, suggesting future applications in the imaging of MMP activity.

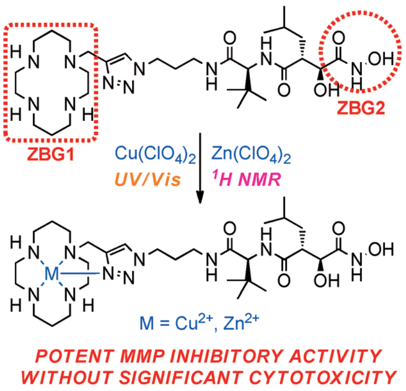

*M. Yu, N. H. Lim, S. Ellis, H. Nagase, J. A. Triccas, P. J. Rutledge,* M. H. Todd**

***99 – 105***

**Incorporation of Bulky and Cationic Cyclam-Triazole Moieties into Marimastat Can Generate Potent MMP Inhibitory Activity without Inducing Cytotoxicity**

© 2013 Wiley-VCH Verlag GmbH & Co. KGaA, Weinheim

*L. P. Wolters, F. M. Bickelhaupt**

***106 – 114***

**Nonlinear d$^{10}$-$ML_2$ Transition-Metal Complexes**

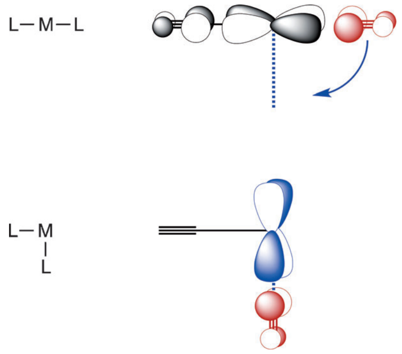

**π Electrons can do that!** They can bend the otherwise linear d$^{10}$-$ML_2$ complexes through backbonding. Bonding analyses reveal that the second π-accepting ligand is, in a sense, hunting for "fresh" (not yet stabilized) $d_\pi$ electrons. The magnitude of the effect depends on the nature of both metal and ligand; L–M–L angles adopt values as small as 128.6°.

*M. Steinmetz, S. Grimme**

***115 – 124***

**Benchmark Study of the Performance of Density Functional Theory for Bond Activations with (Ni,Pd)-Based Transition-Metal Catalysts**

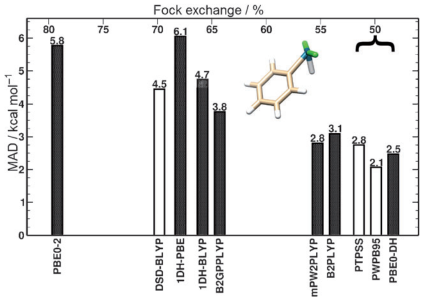

**Catch 23:** An extensive benchmark for evaluating modern density functionals in transition-metal-catalyzed bond-activation reactions is presented. The study focuses on the class of sophisticated double-hybrid functionals and finds dispersion-corrected PWPB95 and PBE0 as the most robust and well-performing methods.

* Author to whom correspondence should be addressed.

Supporting information on the WWW (see article for access details).

This is an open-access article, published under the terms and conditions of the Creative Commons Attribution Non Commercial License.

A video clip is available as Supporting Information on the WWW (see article for access details).

VIP Contributions labeled with this symbol have been judged as "Very Important Papers" by the referees.

### SERVICE

**All the Tables of Contents may be found on the WWW under: http://www.chemistryopen.org**
